# Transcriptomic profiling of the high-vigour maize (*Zea mays* L.) hybrid variety response to cold and drought stresses during seed germination

**DOI:** 10.1038/s41598-021-98907-8

**Published:** 2021-09-29

**Authors:** Heqin Li, Haiwang Yue, Junliang Xie, Junzhou Bu, Li Li, Xueying Xin, Yanming Zhao, Haiyan Zhang, Li Yang, Jianhua Wang, Xuwen Jiang

**Affiliations:** 1 Maize Research Institute/College of Agronomy, Qingdao Agricultural University, Qingdao, 266109 China; 2 Dryland Farming Institute, Hebei Academy of Agriculture and Forestry Sciences, Hengshui, 053000 China; 3 Seed Science and Technology Research Center, China Agricultural University, Beijing, 100193 China; 4 Taocheng Branch of Natural Resources and Planning Bureau of Hengshui City, Hengshui, 053000 China

**Keywords:** Molecular biology, Plant sciences

## Abstract

Abiotic stresses, including cold and drought, negatively affect maize (*Zea mays* L.) seed field emergence and later yield and quality. In order to reveal the molecular mechanism of maize seed resistance to abiotic stress at seed germination, the global transcriptome of high- vigour variety Zhongdi175 exposed to cold- and drought- stress was analyzed by RNA-seq. In the comparison between the control and different stressed sample, 12,299 differentially expressed genes (DEGs) were detected, of which 9605 and 7837 DEGs were identified under cold- and drought- stress, respectively. Functional annotation analysis suggested that stress response mediated by the pathways involving ribosome, phenylpropanoid biosynthesis and biosynthesis of secondary metabolites, among others. Of the obtained DEGs (12,299), 5,143 genes are common to cold- and drought- stress, at least 2248 TFs in 56 TF families were identified that are involved in cold and/or drought treatments during seed germination, including bHLH, NAC, MYB and WRKY families, which suggested that common mechanisms may be originated during maize seed germination in response to different abiotic stresses. This study will provide a better understanding of the molecular mechanism of response to abiotic stress during maize seed germination, and could be useful for cultivar improvement and breeding of high vigour maize cultivars.

## Introduction

Maize (*Zea mays* L.) is an important grain-forage and energy crop, and also an important genetic model plant. The growth and development of maize is highly dependent on good environmental conditions^1^. However, in recent years, various types of abiotic stresses occur frequently in agricultural production, such as low temperature, drought and salinity^2^. These major abiotic environmental stresses seriously affect crop growth and development, especially the normal germination and emergence of seeds, which limits the worldwide agricultural production and development^3^. Abiotic stresses have become an important reason for more than 50% of the global crop yield reduction^4–6^. In China, spring maize is often affected by low temperature and cold damage, and 60% of maize grows in arid areas, which causes 20–30% of maize yield loss every year^1^. Maize seeds are very sensitive to stress in the germination stage, and are prone to emergence difficulties, weak seedlings and other issues, which directly affect agricultural production and cause huge economic losses^7^. Abiotic stresses can cause physiological, molecular and biochemical changes in plants, including damage to cell membrane integrity, inhibition of metabolic function, interference with seed germination and seedling emergence, and even cause plant death^2,6^.

Seed vigour is an important index of seed quality which determine the potential for rapid, uniform emergence and development of normal seedlings under a wide range of field conditions^8^. High vigour seeds are the basis of ensuring crop yield and quality^8–10^. There are many factors affecting seed vigour, but heredity plays a key role. Some important genes have been reported to respond to abiotic stresses such as cold, drought and salt during seed germination^11–13^. High vigour seeds have evolved complex resistance mechanisms to cope with various stress factors and ensure seed germination^8–10^. To improve seed vigour, it is important to understand how plants perceive these stress signals during seed germination. The responses to abiotic stress during seed germination are mainly reflected in cell metabolism and gene expression regulation.

Significant advances have been made in the molecular mechanisms of plant responses to abiotic stresses. Abscisic acid (ABA), gibberellins (GAs), auxins (IAA), ethylene (ETH), cytokinins (CKs) and brassinosteroids (BRs) and other plant hormones play important roles in regulating seed germination under abiotic stresses, such as the GA20ox gene is down-regulated of maize seedling in response to cold stresss^14^. In particular, the mechanism of abscisic acid synthesis, signal transduction and transport is the key to understand the ability of plants to resist abiotic stresses^15,16^. These plant responses to abiotic stresses at the molecular level are mainly regulated by genes regulating the synthesis of osmoprotectants and transporters as well as encoding regulatory proteins such as protein kinases, phosphatases and transcription factors (TFs)^2,17^. TFs are extensively involved in the process of abiotic stress signaling and the responses in plant, and the functions of TFs in plant stress resistance has received extensive attention^18^. Many TF genes from AP2/IREP, bZIP, MYB, NAC and WRKY families have been found to play important roles in various abiotic stresses, and some TF genes have also been proved to be able to improve the stress resistance of model and crops plants^16^. Among them, C-repeat binding transcription factor (CBF) expression was induced only under cold stress in Arabidopsis^19^. OsCTZFP8, a C2H2 zinc finger protein transcription factor, which is induced by a variety of abiotic stresses in rice, and is strongly induced by cold^20^; MAPK (mitogen-activated protein kinase) related genes play an important role in cold and drought stresses signal transduction in maize plant^21^. Increasing maize seed vigour to enhance plant resistance to abiotic stress is considered to be an important measure to transform improved varieties into productivity^4^. However, the molecular mechanism of stress resistance in maize seed germination under abiotic stress is still unclear.

**Figure 1:**
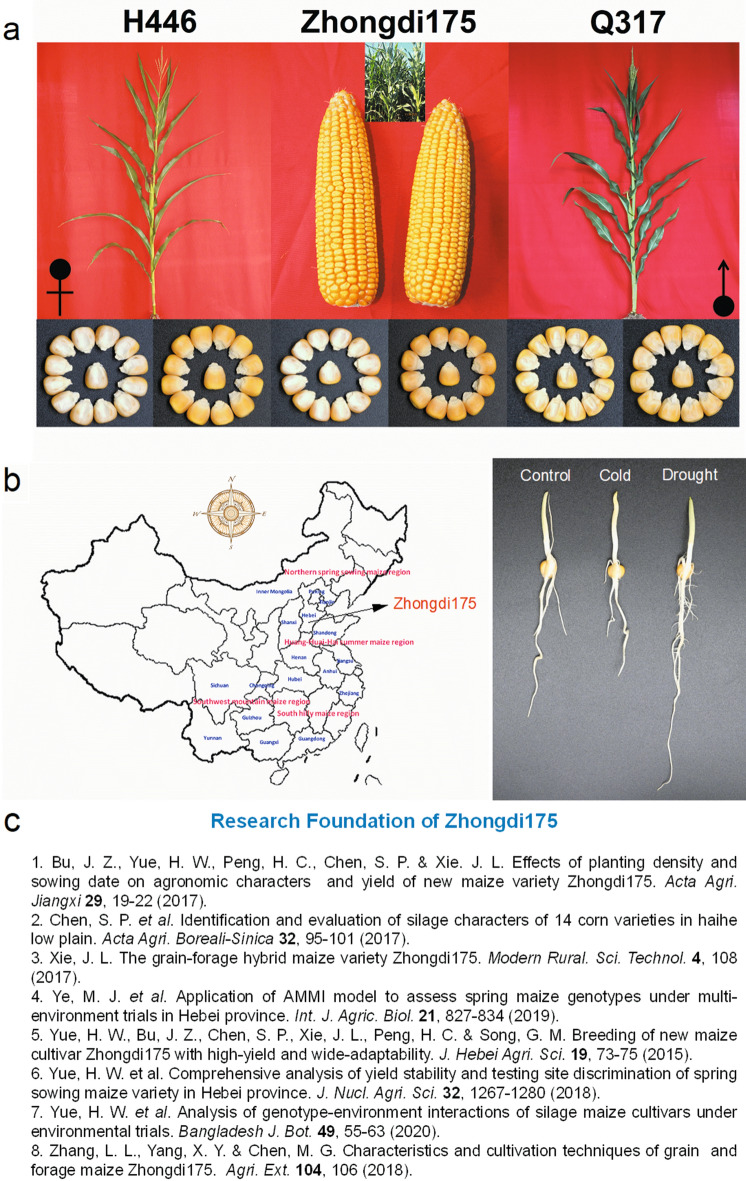
Introduction of Zhongdi175. (**a**) Morphology of the plant, its parent plant, cob and seed of maize variety Zhongdi175. (**b**) The characteristics of Zhongdi175 variety and its seedling morphology under cold and drought stresses during seed germination. (**c**) The literature in the figure is part of the research work carried out by us in the breeding and popularization of Zhongdi175.

Thousands of genes and multiple metabolic and signaling pathways have been found to be involved in abiotic stress responses in plants. Some of these genes and signaling pathways have been shown to play an important role in improving plant tolerance to cold, drought, salt and other stresses^18,22^. Clarifying the molecular mechanism of maize seed germination in response to low temperature, drought and other stresses will help to cultivate high vigour maize varieties and develop new technologies to improve seed vigour. RNA-Seq is a powerful sequencing-based technology, which is particularly suitable for the study of complex gene regulatory networks^23^. To fully understand the molecular response mechanism of maize seed germination (seedling establishment stage) under the cold and drought stresses, the transcriptome profiles of high-vigour cultivar Zhongdi175 (Fig. 1) were obtained at the genome-wide level by using RNA-seq. The differentially expressed genes (DEGs) under cold or/and drought were identified. These data will help to understand and explore the genetic and molecular mechanism of stress response in maize seed germination, and provide gene resources and strategies for maize variety improvement and breeding of high vigour varieties.

## Materials and methods

### Plant materials and stress treatments

Zhongdi175 (H446 × Q317) is a common maize cultivar in Huang-Huai-Hai summer maize area in China, which has developed a widespread reputation for outstanding the characteristics of strong stress resistance, high and stable yield, wide adaptability and so on (Fig. 1). H446 as its female parent is form elite inbred line Zheng58 hybridization with foreign hybrid X1132 then continuous self-cross from seven generations of breeding under alternate between water and drought selecting environmental with many characteristics, ie stress tolerance, flourishing root system, dry resistance, and high yield's seed production. Q317 as Zhongdi175’s male parent is form (H21 × 92–8) × 598 then continuous self-cross multi-generation of breeding with the characteristics of disease-resistant, plant-type compact, high quality and high combining ability. The paternal and maternal traits of Zhongdi175 are complementary and have obvious advantages, especially the previous research indicated that Zhongdi175 is also a representative cultivar of high vigour maize in China (Fig. 1).

In this study, the seeds of Zhongdi175 were provided by Beijing Zhongdi Seed Technology Co. Ltd. and stored in a seed bank (Beijing Kulan Technology Co., Ltd., Beijing, China) at 10 °C, 40% relative humidity (RH). Enough seeds with uniformity and good health were selected for surface sterilization in 1% NaClO (w/v, Beijing Chemical Reagent Company, China) for 10 min, taking out and washing the soaked seeds with distilled water for three times. Two pieces of germination paper which were supplied by Anchor Paper Co., USA, were stacked, thoroughly moistened with distilled water, and then remove the excess water from the paper with a towel. The sterilized seeds were alternately placed in a loose roll of vertical germinating paper (Anchor Paper Co., USA) and incubated in an artificial climate incubator (GXZ-380C) at 25 ± 0.5 °C (12-h light/12-h dark), with nine replicates of 100 seeds per replicate. After four days of seed germination, different treatments were carried out, and untreated seeds/replicates were used as control. The treatments are as follows: (1) cold treatment: three replicates were germinated at 4 ± 0.5 °C (12-h light/12-h dark) for 48 h; (2) drought treatment: seeds of three replicates were transferred to a paper bed containing PEG6000 (20%) solution, then were germinated at 25 ± 0.5 °C (12-h light/12-h dark) for 48 h; (3) control: three replicates continued to germinate for 48 h at 25 ± 0.5 °C (12-h light/12-h dark). For each replicate, ten seedlings with uniformity were randomly selected for RNA-seq analysis, and the other seedlings were used for physiological indices measurement. All the samples were stored in ultra-low temperature refrigerator (DW-86L 388J) at − 80 °C.All applicable international, national, and institutional guidelines for the use of plants in the present study were followed.

### RNA-seq analysis and sequence assembly

The Illumina HiSeq™ 2500 plant form was applied for RNA-seq analysis by Gene Denovo Biotechnology Co. (Guangzhou, China). To obtain a comprehensive overview of Zhongdi175 transcriptome, nine libraries were constructed and pariedend sequencing was carried out. In brief, the mRNA was enriched by Oligo (dT) magnetic beads and the cDNA fragments were purified by QIAquick PCR extraction kit, end repaired, poly (A) was added and ligated to that Illumina sequencing adapter. The clean data were obtained by removing adapters, low quality reads containing low quality (Q-value ≤ 20) bases from the raw data. All clean sequencing data were deposited in the NCBI's Sequence Read Archive (SRA, https://www.ncbi.nlm.nih.gov/sra) under accession number PRJNA533857.

Bowtie2 was used to map readings to a ribosome RNA (rRNA) database^24^. TopHat2 (version 2.1.1) was used to map the rRNA removed reads of each sample to reference genome (B73_RefGen_v4)^25^. The reconstruction of transcripts was carried out with software Cufflinks^26^, which together with TopHat2, were used to identify new genes and known genes. All the reconstructed transcripts were aligned to the reference annotation by using Cuffcompare. Transcripts with nucleotide more than 200 bp and exon numbers more than two were defined as novel genes. The flow chart of bioinformatics analysis see Supplementary Figure S1.

### Functional annotation and classification of transcripts

BLASTX analysis was used to analyze gene function annotation with the non-redundant (Nr) protein sequence database at GeneBank (http://www.ncbi.nlm.nih.gov), Swiss-Prot (http://www.expasy.ch/sprot) and KEGG (http://www.genome.jp/kegg). The significant threshold for E-value was set to ≤ 10^−6^. Gene Ontology (http://www.geneontology.org) was employed to get GO enrichment based on biological process, cellular component features, and molecular function, with FDR ≤ 0.05 as a threshold. All genes were blasted against the plantTFDB (http://planttfdb.cbi.edu.cn) with a cutoff E-value ≤ 1e^−5^ to identify putative transcription factors (TFs).

### DEGs identification

To identify DEGs across treatments, the edgeR package (http://www.r-project.org/) was used. Cleaning reads of each library were rearranged with reference genes using Bowtie software, and the number of mapped reads was calculated by RSEM^27^. The gene expression level was normalized by using fragments per kilobase million (FPKM) method, and the effect of different gene lengths and sequencing data amount on the calculation of gene expression were eliminated^26^. The genes from at least one FPKM ≥ 1 treatment were used for further analysis. The fold change of each gene under cold or drought condition was determined by comparing the FPKM value with that of the control, and the genes with fold change ≥ 2 and false discovery rate (FDR) < 0.05 in the comparison were taken as significant DEGs by edgeR package^28^.

### qRT-PCR analysis

A total of eight genes with different expression patterns in our Illumina RNA-seq data were randomly chosen to further verify by qRT-PCR. First strand cDNA synthesis and qRT-PCR were performed using PrimeScript™ RT reagent kit with gDNA Eraser (Perfect Real Time) (Takara, Dalian, China) and SYBR® Premix Ex Taq™ II (Tli RNaseH Plus, Shiga, Japan) (TaKaRa), respectively. The reaction was performed on the BIO-RAD CFX96 sequence detection system. The specific primers (Supplementary Table S1) of eight genes were designed with online Integrated DNA Technologies (https://sg.idtdna.com/scitools/applications/realtimepcr/). And ZmActin was used as an internal reference gene. Each PCR reaction (20 µL) contained 10 µL of SYBR® Premix Ex Taq™ II, 10 µM of forward and reverse primers, and 2 µL of template cDNA which diluted 10 folds with deionized water. Three independent biological replications were performed for each sample. Relative expression levels were calculated using the 2^−∆∆CT^ method^29^. And the regression coefficient between qRT-PCR results and RNA-seq data was analyzed using IBM SPSS Statistics for Windows Version 19.0 (IBM Corp.).

## Results

### Illumina sequencing, De novo assembly and functional annotation of the Zhongdi175 transcriptome

To comprehensively understand the effects of cold and drought stresses on gene expression of Zhongdi175 during seed germination, the total RNA of seedlings was sequenced by Illumine system. We performed transcriptomics analysis of maize seeds from control, cold and drought environments to investigate the response of plants to abiotic stresses during seed germination, with three replications per environment. Clean data of 35,743,617,000 bp (control), 38,835,975,900 bp (cold) and 35,041,415,400 bp (drought) were obtained. After the adapters, more than 10% of the unknown nucleotides and more than 50% of the low quality (Q-value ≤ 20) bases were removed, a total of 234,397,256 (control), 253,824,644 (cold) and 229,851,862 (drought) high quality clean reads (HQ Clean Reads) were used for assembly (Table 1). The Q30 scores of all libraries were above 92%, with an average of 94.17%, indicating that the sequencing results were reliable. On average, 78.44% of the reads were mapped to the maize reference genome sequence (B73_RefGen_v4) using the Tophat2 default parameter (Table 1). Transcriptome assembly was carried out using the cufflinks software and 32,269 known and 2358 new genes were gotten, and the proportion of known genes and new genes were 93.2% and 6.8%, respectively (Supplementary Table S4). Based on the blast search against the PlantTFDB, a total of 2248 genes of 56 TF families were identified as transcription factors (Table 2 and Supplementary Table S5).

**Table 1:** Overview of the transcriptome sequencing and assembly.

Sample	Clean data (bp)	HQ Clean data (bp)	Q30 (%)	Clean reads	HQ clean reads (%)	Total reads	Mapped reads
Control-1	11,816,952,600	11,490,594,791	95.24	78,779,684	98.49	74,283,466	57,160,868
Control-2	11,454,157,500	11,082,646,782	94.25	76,361,050	98.17	71,979,126	54,524,130
Control-3	12,472,506,900	12,107,959,846	94.02	83,150,046	98.43	77,841,374	60,324,281
Cold-1	13,223,859,000	12,763,322,032	94.11	88,159,060	97.94	82,243,428	65,109,690
Cold-2	14,408,499,900	14,009,146,352	93.39	96,056,666	98.47	93,090,796	73,822,273
Cold-3	11,203,617,000	10,714,718,664	92.06	74,690,780	97.59	70,994,574	55,550,746
Drought-1	12,659,780,700	12,301,990,225	94.04	84,398,538	98.39	81,167,504	64,323,556
Drought-2	11,057,785,800	10,741,036,029	95.12	73,718,572	98.34	70,856,384	56,616,031
Drought-3	11,323,848,900	11,016,930,640	95.27	75,492,326	98.45	72,247,230	57,734,732

*Numbers 1, 2 and 3 indicate the three biological replicates.

**Table 2:** Transcription factor (TF) gene families identified in the Zhongdi175 transcriptome.

TF family	Number of genes	TF family	Number of genes
MYB	203	NF-YB	20
bHLH	196	CO-like	19
ERF	165	WOX	19
WRKY	132	GRF	18
bZIP	129	DBB	17
NAC	123	NF-YC	16
C2H2	120	HB-other	15
GRAS	74	NF-YA	13
HD-ZIP	62	Nin-like	13
G2-like	61	YABBY	13
FAR1	58	CPP	12
Trihelix	60	E2F/DP	12
C3H	56	SRS	11
MYB_related	56	ARR-B	10
Dof	48	CAMTA	9
MIKC	48	EIL	9
B3	47	BES1	6
ARF	40	HB-PHD	6
LBD	38	LSD	6
AP2	34	RAV	5
GATA	34	BBR-BPC	4
SBP	34	VOZ	4
TCP	34	NF-X1	3
HSF	31	LFY	2
TALE	31	S1Fa-like	2
GeBP	23	Whirly	2
ZF-HD	22	HRT-like	1
M-type	21	STAT	1

A total of 2248 TF genes from 56 TF families were identified.

### Identification and comparison of stress-specific differentially expressed genes (DEGs)

**Figure 2:**
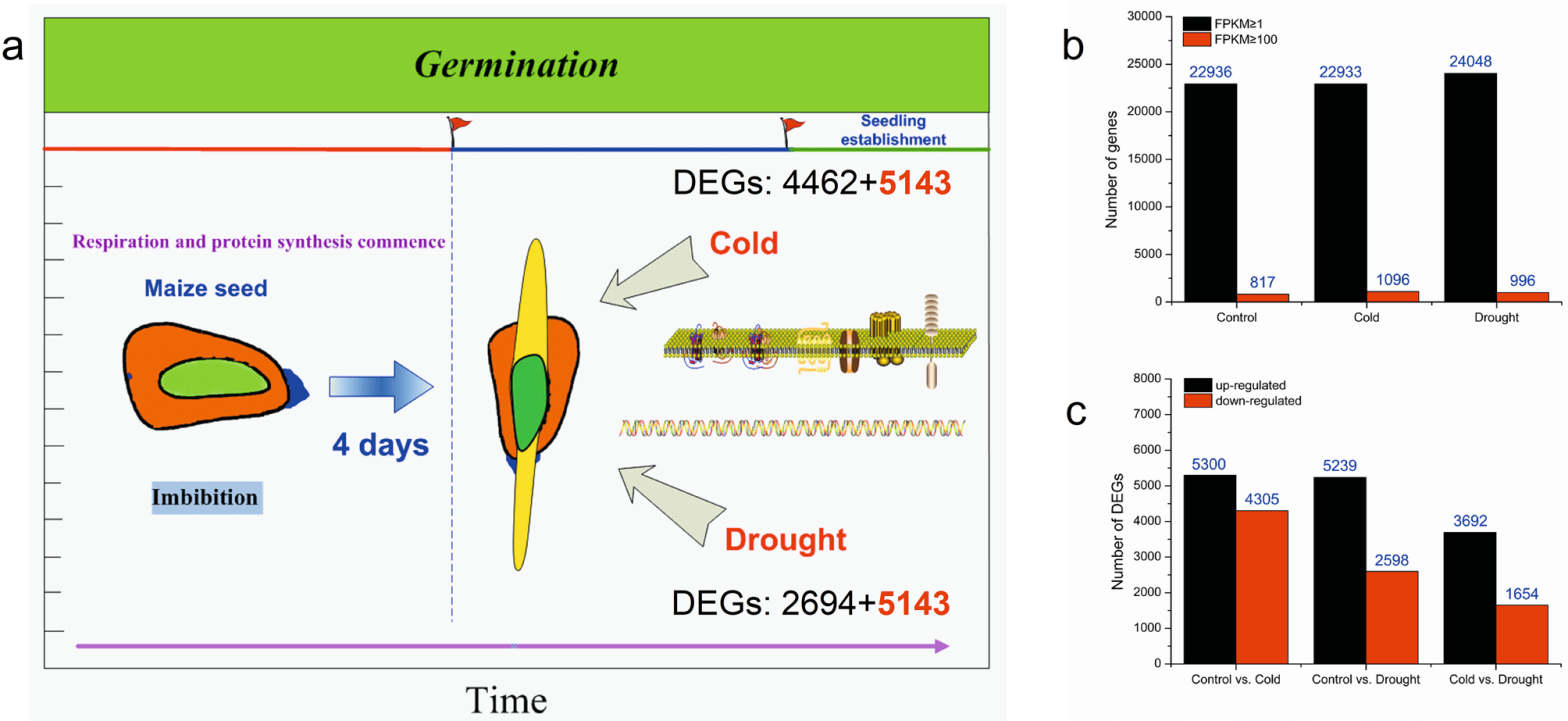
DEGs of different treatments of Zhongdi175. (**a**) Schematic diagram of stress during seed germination and venn analysis of DEGs. (**b**) Expression quantification of genes of Zhongdi175. (**c**) The numbers of DEGs up- and down-regulated between different treatments. The software package edgeR (version 3.14.0) (http://www.rproject.org/) was used to identify DEGs.

Seed germination (generalized) includes four stages: imbibition, protrusion, germination and seedling establishment^30^. In seedling establishment, a total of 22,936, 22,933 and 24,048 genes with FPKM ≥ 1 were selected from the sample of control, cold and drought, respectively (Supplementary Table S6). Obviously, the number of genes with FPKM ≥ 1 was larger in the samples drought stressed samples than in other samples, which may be the reason why drought stress increased transcript variation in samples. However, there were 8,171,096 and 996 genes with FPKM ≥ 100 from the sample of control, cold and drought, respectively (Fig. 2a,b and Supplementary Table S6). Compared with the control, there were more genes with high FPKM value in cold treatment than in drought treatment, indicating that the effect of cold stress on Zhongdi175 was greater than that of drought stress. Each stress sample of Zhongdi175 was compared to the control to determine DEGs (|log2 FC|≥ 1 and FDR < 0.01). A total of 12,299 DEGs were obtained, of which 9605 (5300 up- and 4305 down-regulated) and 7837 (5239 up- and 2598 down-regulated) genes were differentially responsive to cold and drought stresses, respectively, of which 5143 DEGs were common to cold- and drought-stress (Fig. 2a,c and Supplementary Tables S7,S8). We also compared the DEGs under the cold- and drought- stress, and found 3692 up-regulated genes and 1654 down-regulated genes in Zhongdi175 (Fig. 2c and Supplementary Table S9).

### Functional classification of DEGs

**Figure 3:**
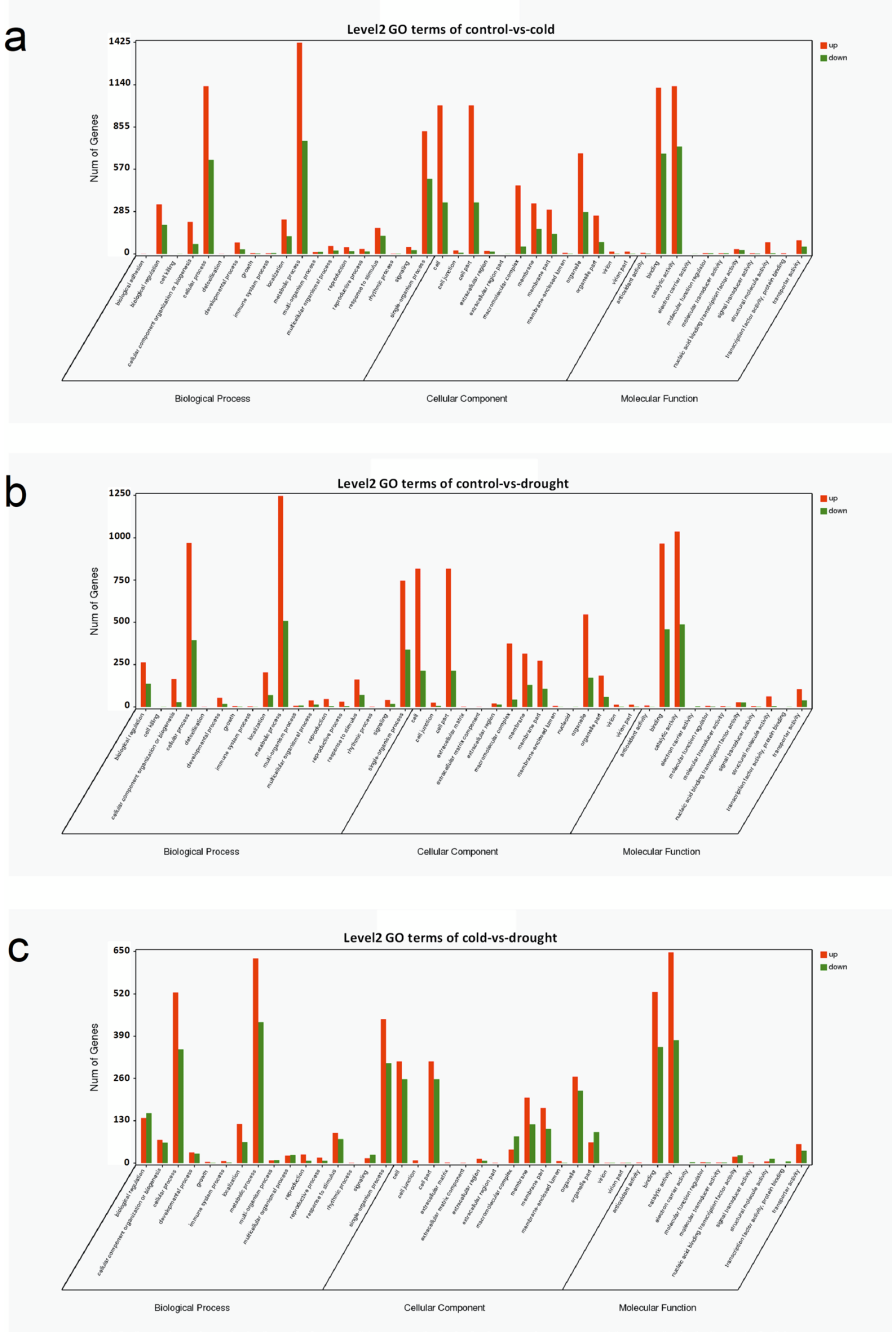
Histogram of GO classification of the annotated genes. (**a**) control vs. cold. (**b**) control vs. drought. (**c**) cold vs. drought. Left Y-axis indicated the numbers of genes in each GO category. Gene Ontology (http://www.geneontology.org) was employed to get GO enrichment.

GO analysis was performed to determine the function of the identified DEGs. Based on FDR < 0.05, 43, 44 and 42 GO terms were overrepresented in cold, drought and between drought and cold, respectively (Supplementary Tables S10–S15). To control versus cold, the “binding”, “catalytic activity”, “cellular process”, “cell”, “cell part”, “metabolic process”, “single-organism process” and “organelle” were mostly dominant (Fig. 3a and Supplementary Table S10). For control vs. drought and cold vs. drought, the mostly dominant terms of them were same to control vs. cold (Fig. 3b,c and Supplementary Tables S10–S15).

**Figure 4:**
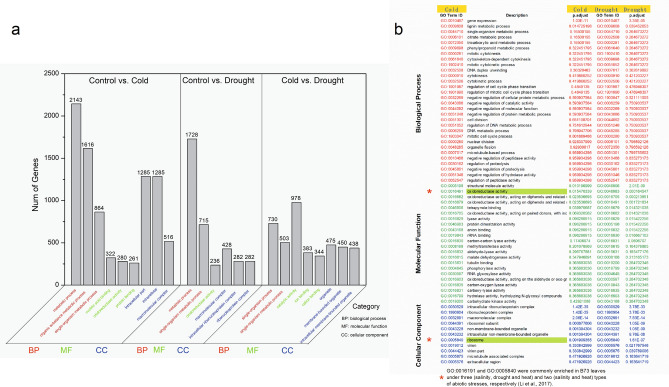
Numbers of differentially expressed genes (DEGs) in main KEGG pathway (**a**), and comparative analysis of enriched GO terms (**b**).

**Figure 5:**
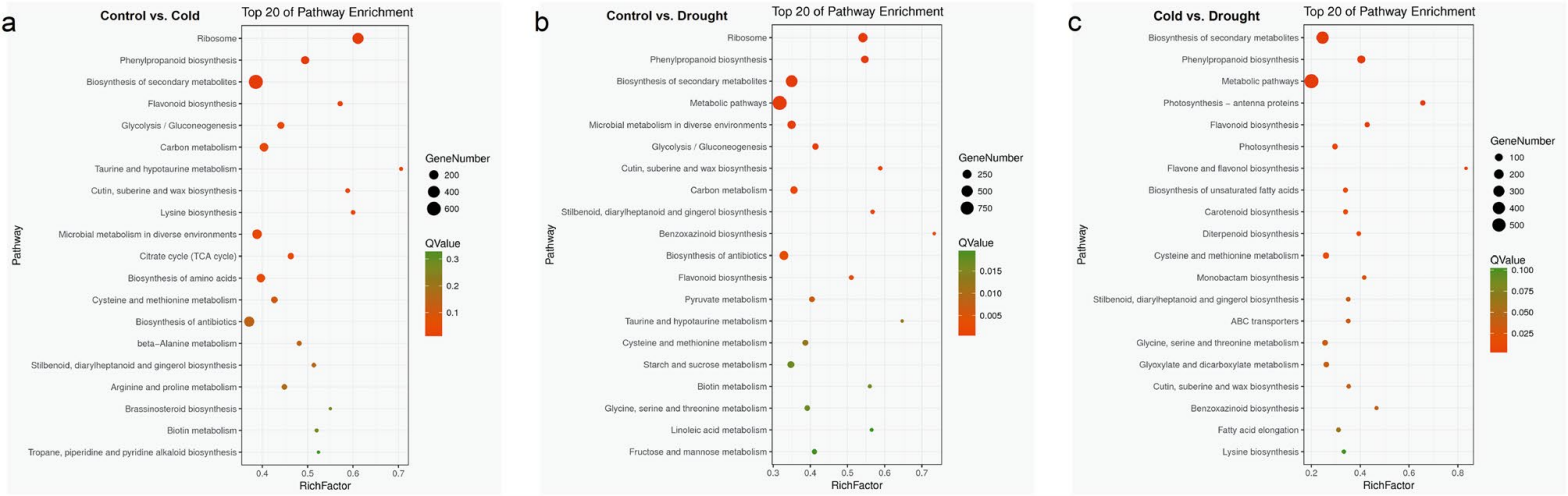
KEGG pathway annotation of differentially expressed genes. The top 20 statistics of pathway enrichment were shown. (**a**) control vs. cold. (**b**) control vs. drought. (**c**) cold vs. drought. The ggplot2 package (http://www.r-project.org/) was used to plot KEGG enrichment analysis.

In the DEGs identified between stress and control samples, 121 and 119 GO terms were enriched in the comparison of cold and drought to control, respectively. To determine the transcriptomic changes that occur in response to various abiotic stresses, the enriched GO terms under different stress conditions were compared, and all commonly enriched GOs were summarized in Fig. 4. 63 GO terms were enriched in all datasets, of which 30, 22, 11 GO terms belonged to biological process, molecular function and cellular component, respectively (Figs. 4 and 5). In addition, GO:0016491 and GO:0005840 were also commonly enriched in B73 leaves under three (salinity, drought and heat) and two (salinity and heat) types of abiotic stresses, respectively^31^. Under cold and drought stresses, many GO terms are closely related to the stress resistance of Zhongdi175 seed germination (Supplementary Tables S2,S10–S15). For example, the expression of genes encoding peroxidase (entrzID_542505), heat shock protein binding protein (entrzID_100280673) and peroxidase (entrzID_103641351, entrzID_103642599, entrzID_103644044, and entrzID_103647239) in seedlings under cold stress was down-regulated during seed germination of Zhongdi175. The expression of genes regulating heat shock protein (entrzID_103625886) and peroxidase (entrzID_103636702) were up-regulated under cold and drought stresses (Supplementary Tables S10–S15).

In order to further investigate the biological function of these DEGs, we performed pathway enrichment analysis using KEGG. The results showed that 2292 DEGs were enriched in 130 pathways of control vs. cold samples, 1866 were enriched in 128 pathways of control vs. drought samples, and 1048 were enriched in 127 pathways of cold vs. drought samples (Supplementary Tables S16–S18). Compared with drought stress, more DEGs were enriched in germination metabolic pathway of Zhongdi175 seeds under cold stress. It is worth noting that “ribosome”, “phenylpropanoid biosynthesis”, “biosynthesis of secondary metabolites” and “flavonoid biosynthesis” pathways were significantly enriched in the control vs. cold comparison; the “ribosome”, “phenylpropanoid biosynthesis”, “biosynthesis of secondary metabolites” and “metabolic pathways” pathways were significantly enriched in maize plants in response to drought stress during Zhongdi175 seeds germination. Moreover, the “biosynthesis of secondary metabolites”, “phenylpropanoid biosynthesis”, “metabolic pathways” and “photosynthesis—antenna proteins” pathways were significantly enriched in the cold vs. drought comparison (Fig. 5).

### Identification of abiotic stress responsive transcription factors

Additionally, the DEGs encoding the transcription factor (TF) was analyzed. A total of 2248 DEGs encoding cold and drought stress responsive transcription factors (TFs) were detected during Zhongdi175 seeds germination, and these transcription factors belonged to 56 transcription factor families respectively. Most of the identified DEGs encoded members of the bHLH, bZIP, C2H2, ERF, GRAS, MYB, NAC and WRKY related TF families (Table 2). The MYB family was the largest TF family responding to abiotic stress, with 203 DEGs. A total of 196, 165, 132, 129, 123, 120, and 74 DEGs were identified, belonging to the bHLH, ERF, WRKY, bZIP, NAC, C2H2 and GRAS families respectively. Expression of most MYBs and bHLH was up-regulated; conversely, WRKY was typically down-regulated. For example, the expression of MYB (entrzID_100285825 and entrzID_103634904) and bHLH (entrzID_103631776) was up-regulated and WRKY (entrzID_100279570, entrzID_100281558, and entrzID_100285217) was down-regulated under cold and drought stresses (Supplementary Table S5). The different expression patterns of TFs in maize seedlings under cold and drought stress indicated that Zhongdi175 had a wide range of abiotic stress resistance mechanisms during seed germination.

### Expression of hormone biosynthesis and signal transduction genes under abiotic stress

**Figure 6:**
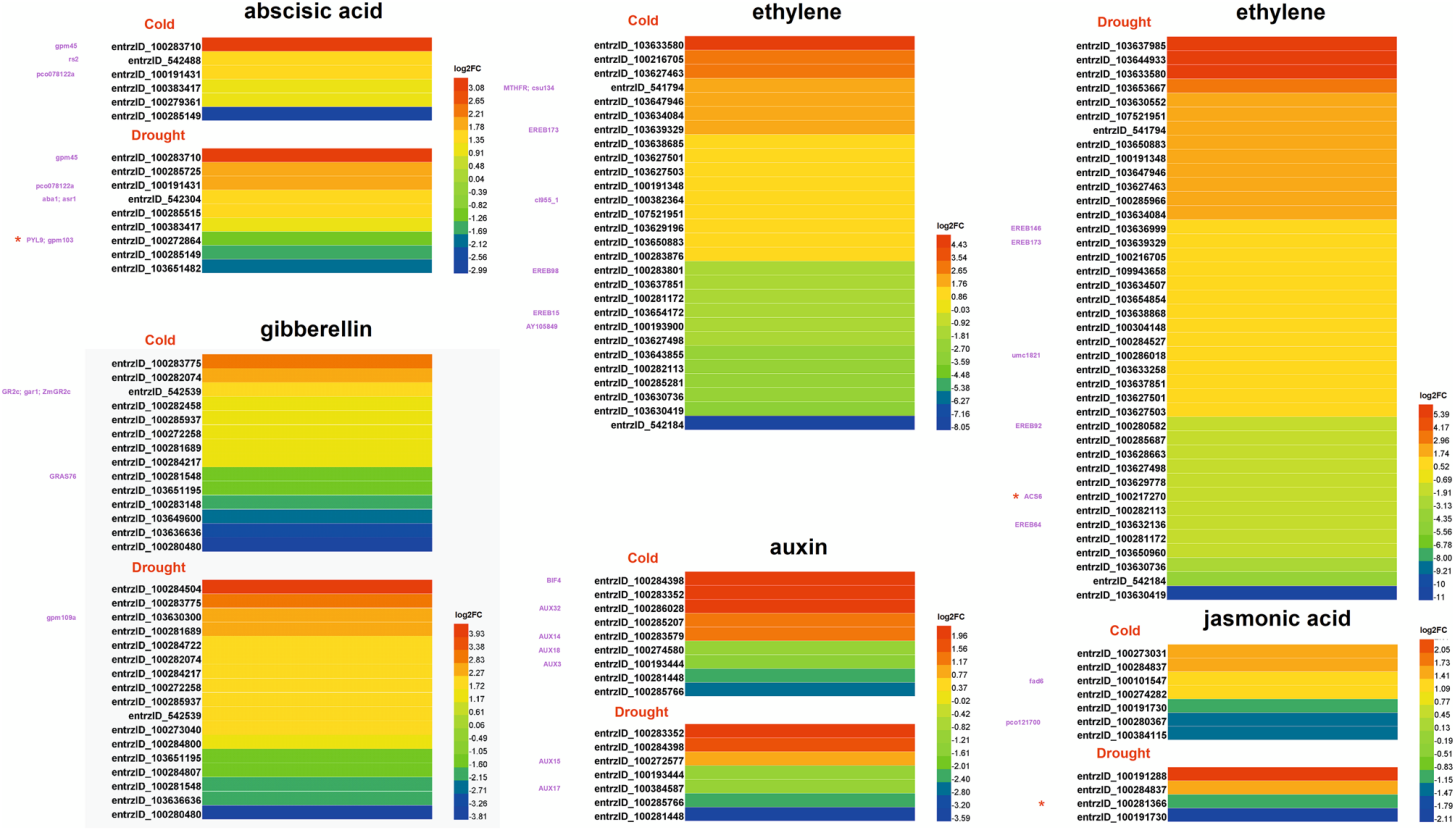
Heatmap of DEGs involved in hormone biosynthesis and signal transduction pathways in two pairwise comparisons of stresses against control. The software of Heml (Heatmap illustrator, version 1.0) was used for heatmap analysis.

Approximately, 70 DEGs involved in hormone biosynthesis and signal transduction pathways have been identified, such as the abscisic acid (ABA), jasmonic acid (JA), ethylene (ET), auxin (IAA), and gibberellin acid (GA) pathways. In addition, in comparison between stress and control samples, four DEGs (entrzlD_100283710, entrzlD_100191431, entrzlD_100383417 and entrzlD_100285149) encoding ABA biosynthesis and catabolism enzymes as well as ABA receptors were obtained (Fig. 6). During the germination of Zhongdi175 seeds, the expression levels of most ABA biosynthetic genes were up-regulated. 9-cis-epoxycarotenoid dioxygenase (NCED) is the rate-limiting enzyme in the biosynthesis of ABA. Four up-regulated NCEDs were identified, including XLOC_046985 and entrzID_100501444 in cold stress, entrzID_100501454 in drought stress, and entrzID_732819 in both cold and drought stresses. We also detected 7 (JA), 28 (ET), 9 (IAA), 14 (GA) DEGs in cold stress and 4 (JA), 40 (ET), 7 (IAA), 17 (GA) in drought stress DEGs involved in phytohormone biosynthesis pathways, including some important gene family genes. For example, genes encoding auxin-responsive Aux/IAA family member (entrzID_100283579 and entrzID_100286028), were up-regulated under cold stress; gibberellin 20 oxidase (entrzID_100283148), auxin-responsive Aux/IAA family member (entrzID_100274580), were down-regulated under cold stress; gibberellin 2-beta-dioxygenase (entrzID_100273040), gibberellin 20 oxidase (entrzID_100284800), were up-regulated under drought stress; however, auxin-responsive Aux/IAA family member (entrzID_100193444 and entrzID_100285630), gibberellin 2-beta-dioxygenase (entrzID_100280480), were down-regulated under cold and drought stresses (Fig. 6; Supplementary Tables S7–S9). During seed germination, the expression of most of these phytohormone biosynthesis related genes were up-regulated in Zhongdi175 seedlings, but their expression levels were significantly affected by stress conditions. In addition, entrzlD_100272864, entrzlD_100217270 and entrzlD_100281366 were commonly enriched in B73 leaves under abiotic stresses (red stars in Fig. 6)^31^. These results suggest that the biosynthesis of phytohormones is reprogrammed under different abiotic stresses.

### Flavonoid metabolism and response to abiotic stresses

**Figure 7:**
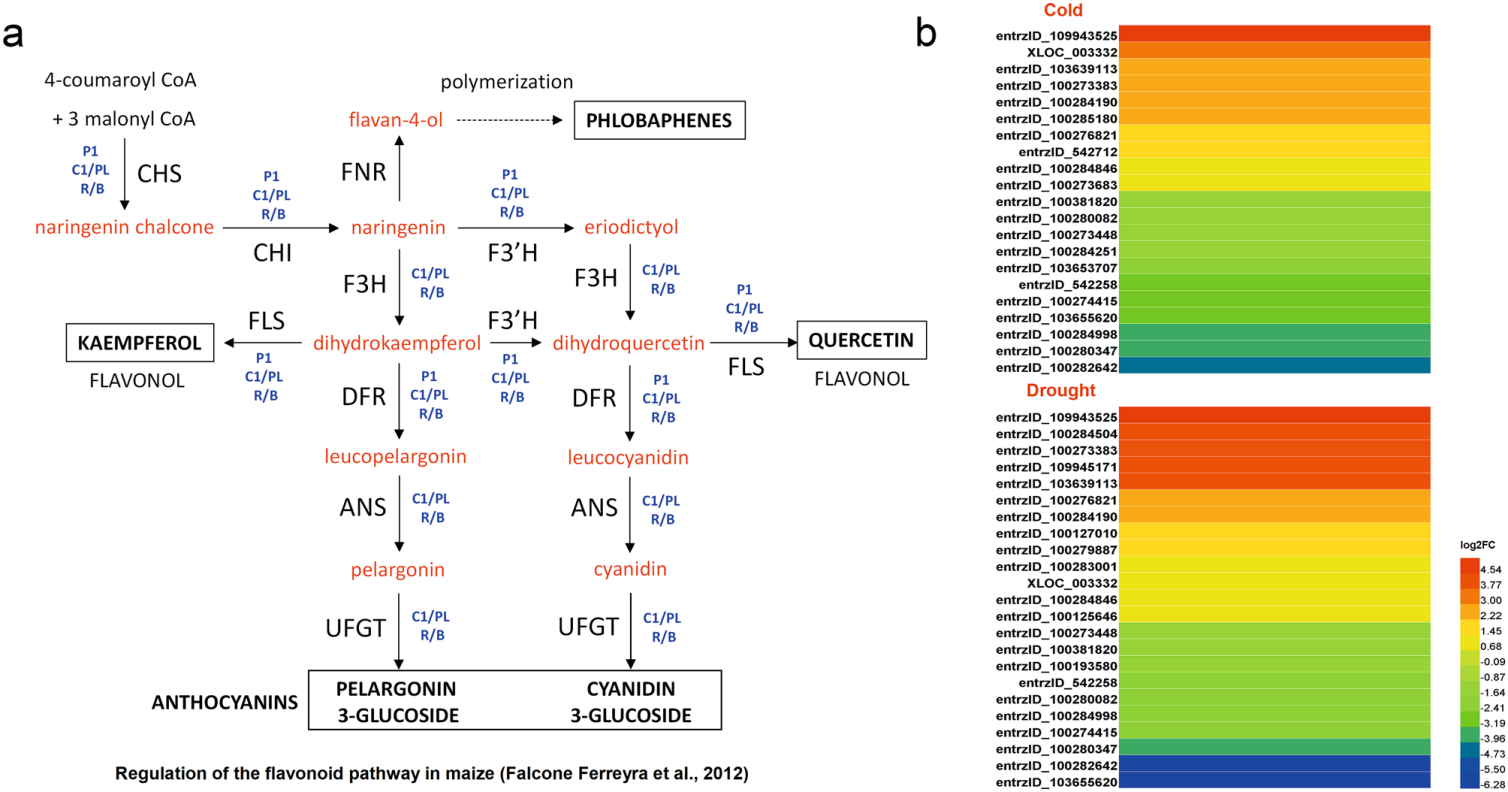
Regulation of the flavonoid pathway in maize (**a**), and the heatmap of DEGs involved in flavonoid metabolism pathways in two pairwise comparisons of stresses against control (**b**).

Plants produce a myriad of specialized metabolites to abiotic stresses. Flavonoids are a large class of important plant polyphenolic secondary metabolites, which have antioxidant, anti-ultraviolet, anti-plant pathogens and other physiological function^32,33^. In this study, 21 (cold stress) and 23 (drought stress) DEGs in flavonoid metabolism pathways were identified (Fig. 7), of which 15 were common genes. Among the 15 genes, 7 genes were up-regulated and 8 genes were down-regulated under cold and drought stresses. Two genes (entrzID_100381820 and entrzID_542258) encoding phenylalanine ammonia-lyase, two genes (entrzID_100274415 and entrzID_100282642) encoding chalcone synthase and one gene (entrzID_100284998) encoding were identified *trans*-cinnamate 4-monooxygenase, which were mainly down-regulated by cold and drought stresses. And phenylalanine ammonia-lyase (entrzID_109943525), chalcone isomerase (entrzID_100276821), flavanone 3-hydroxylase (entrzID_542712), flavonoid 3',5'-hydroxylase (entrzID_103639113), were up-regulated under cold and drought stresses. In addition, the expression of the gene encoding caffeoyl-Coa methyltransferase, entrzID_100273683, and the gene encoding flavonoid 3-monooxygenase, entrzID_103653707, was up-regulated and down-regulated under low temperature stress, respectively. In particular, we identified an important gene in the flavonoid pathway, entrzID_100127010, encoding leucoanthocyanidin dioxygenase, which was up-regulated under drought stress (Supplementary Tables S7–S9). Moreover, some of the genes identified have also been reported in other studies, such as entrzID_100381820 (Zm00001d017279) encoding phenylalanine ammonia-lyase, which is down-regulated in drought-tolerant maize line YE8112^34^; entrzID_100274415 (GRMZM2G422750) encodes a chalcone synthase that is down-regulated in the ovary by drought in maize inbred line B73 to improve drought tolerance in maize^35^.

### Sucrose metabolism and cell growth promotion response to abiotic stresses

Soluble sugar, as an important osmoprotectants, plays an important role in plant osmotic regulation^35,36^. In this study, the expression of genes encoding galactinol synthase (entrzID_606403 and entrzID_606405), trehalose-phosphate phosphatase (entrzID_100285860), sucrose synthase (entrzID_542091) were up-regulated under both cold and drought stresses; the expression of genes encoding sucrose synthase (entrzID_542365), trehalose phosphate synthase (entrzID_100191265) were down-regulated under both cold and drought stresses (Supplementary Tables S7–S9). Fructokinase (entrzID_542108) and hexokinase (entrzID_100170246) genes were up-regulated in Zhongdi175 seedlings under cold and drought stress, respectively (Supplementary Tables S7–S9).

To cell-wall metabolism related genes, the expression of entrzID_542649 encoding beta-expansin precursor, was up-regulated under drought stress, and entrzID_541807, entrzID_542685 and entrzID_542139 encoding cellulose synthase, were up-regulated under both cold and drought stresses. And the expression of hypocotyl elongation protein ortholog gene (entrzID_100192868) was up-regulated in Zhongdi175 seedlings under cold stress (Supplementary Tables S7–S9).

### qRT-PCR verification

**Figure 8:**
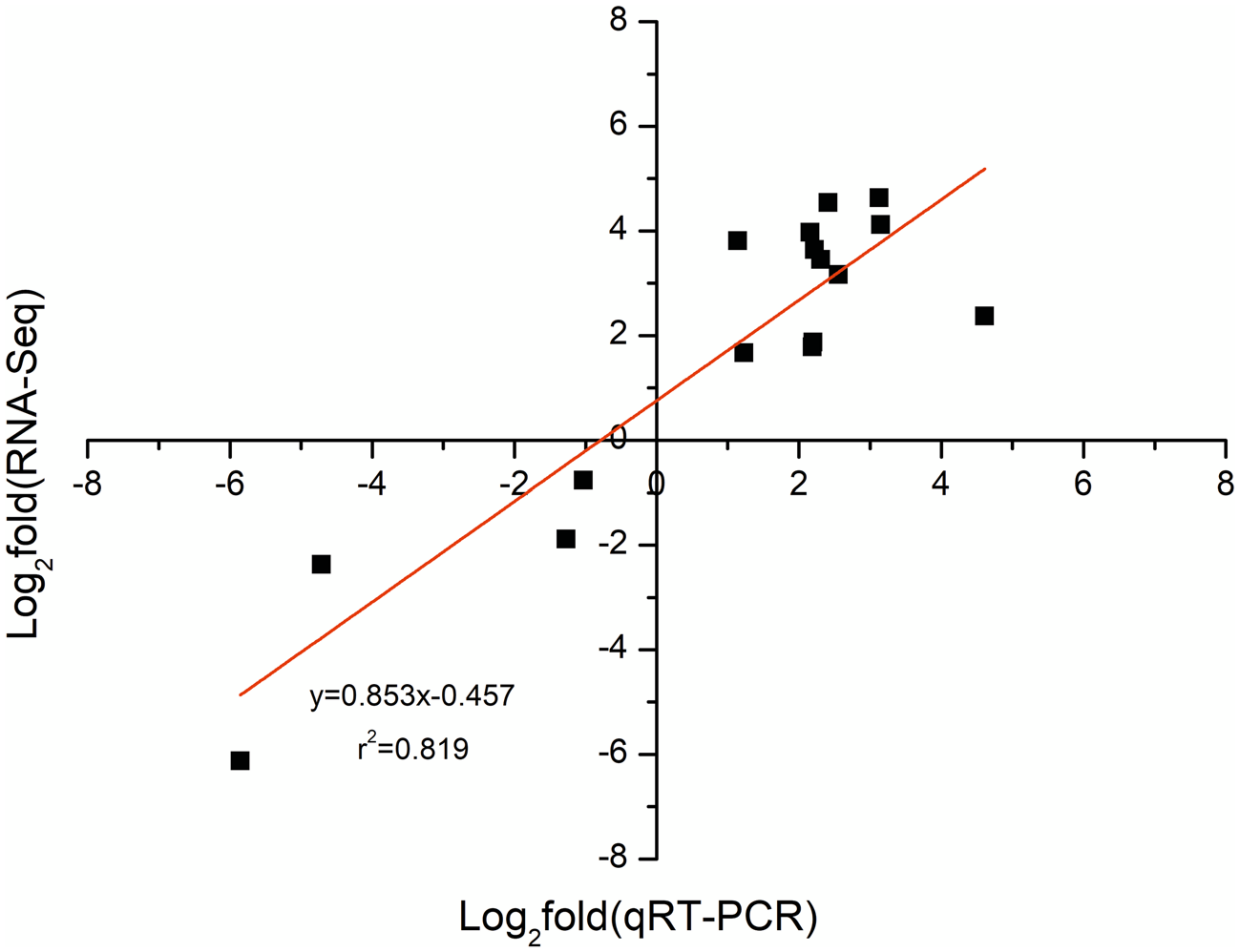
qRT-PCR verifications of DEGs by RNA-seq. The IBM SPSS Statistics for Windows Version 19.0 (IBM Corp.) was used for regression analysis.

Eight DEGs were randomly selected for qRT-PCR analysis of control and stress samples to verify the reproducibility of the gene expression data of Zhongdi 175 seedlings obtained by RNA-seq analysis (Supplemental Table S1). The ratio of expression levels found between stressed samples and controls using qRT-PCR was compared to the ratio of expression measured by RNA-Seq. A significant correlation was observed between RNA-Seq and qRT-PCR data (r^2^ = 0.819, *p* < 0.001, Fig. 8), which confirms the effectiveness of DEGs in this study (Fig. 8). Therefore, the comparison of qRT-PCR and RNA-Seq analysis data of Zhongdi175 seedlings fully verifies the results of our transcriptome study.

## Discussion

Abiotic stresses (such as cold, drought, salt, etc.) seriously affect crop growth, development and yield. Worldwide, abiotic stresses cause major crop yields to drop by more than 50%^4^. Especially, spring sowing maize is often affected by low temperature, drought and other harsh environments. Zhongdi175 is the representative of spring sowing maize varieties with low temperature tolerance and drought resistance in arid and semi-arid areas (Fig. 1). Understanding of the molecular mechanism of Zhongdi175 in response to abiotic stress would be helpful for the breeding of high vigour cultivars. With the development and maturity of high-throughput transcriptome sequencing technology, RNA-seq technology has been successfully applied to transcriptome or genome-wide analysis of rice^37^, maize^38,39^, wheat^40^ and other crops. In particular, this study may help us to better understand the molecular basis of maize seed germination response to abiotic stress.

### Overview of Zhongdi175 transcriptome

Seed germination (generalized) includes four stages: imbibition, protrusion, germination and seedling establishment^30^. In this study, we selected the seedling establishment of seed germination, because the germination stage of maize is particularly sensitive to abiotic stress^15^. A total of 32,269 known and 2358 new genes were identified by transcriptomic analysis in maize seedling during seed germination under different conditions (Supplementary Table S4). Under cold stress (4 °C), 5300 and 4305 DEGs were up-regulated and down-regulated, respectively (Supplementary Table S7). And the changes of gene expression at the whole transcriptome level indicated that the gene expression was up-regulated (5239) and down-regulated (2598) under drought stress (Supplementary Table S8). Here we performed comparative transcriptome profiling of Zhongdi175 in response to cold and drought stresses, and found that an activation of gene network was involved during seed germination. In particular, 5143 co-regulated DEGs by two stresses, 4462 specific to cold stress, 2694 specific to drought stress, were identified, and this results revealed that there were common and different molecular mechanisms of maize seed germination response to cold and drought stresses. The results are similar to those found in *Arabidopsis*^41^, rice^42^, wheat^43^ and maize^15,44^. However, compared with the results obtained by Lu et al. (2017), more DEGs were found in this study, which may be caused by different maize varieties and different growth stages. The co-regulated DEGs belongs to a subset of key genes for cold and drought resistance in maize seed germination, which may play an important role in the adaptation to stress during maize seed germination. Based on the functional annotation of DEGs, the responses of maize seed germination to abiotic stresses were revealed to involve multiple biological pathways, such as hormone metabolism and signal transduction, transcriptional regulation and flavonoid metabolism.

### DEGs encoding transcription factors

Transcription factors (TFS) play an important role in stress signal transduction pathways, which regulate the expression of specific stress-responsive genes in plants^22,45^. Major plant TF families, including MYB, NAC, and WRKY, as important regulatory factors in plant response to various abiotic and biotic stresses, have attracted much attention^12,46,47^. In this study, at least 2248 TFs in 56 TF families were identified that were involved in drought and/or cold treatments during seed germination. According to the number of genes in each TF family identified, the top eight TF families in order were MYB, bHLH, ERF, WRKY, bZIP, NAC, C2H2, and GRAS (Table 2). Different members of these families were up-regulated or down-regulated under cold or/and drought stress, such as ARF, bHLH, bZIP, GRAS, MYB, NAC, WRKY. The MYB TF family was the largest class in response to abiotic stress in Zhongdi175 seed germination. MYBs involve in regulating secondary metabolism and cell morphology, and are response to biotic and abiotic stresses^46,48^, some of which regulate flavonoid biosynthesis and involve in abiotic stress responsiveness in tobacco^49,50^. Here, entrzID_100285825, entrzID_103634904, entrzID_100101513, entrzID_100194332 and entrzID_103643223 encoding MYB, were regulated by cold and drought stresses (Supplementary Tables S5,S7–S9), of which entrzID_103634904 (Zm00001d008808) and entrzID_100194332 (Zm00001d011297) were up-regulated in the drought tolerance of maize inbred line YE8112^34^, suggesting the two MYB TFs were important drought responsive genes for maize. Moreover, members of bHLH (entrzID_103631776), WRKY (entrzID_100280700), were up-regulated under both stresses; bHLH (entrzID_103635409), NAC (entrzID_100273749 and entrzID_100383937), NF-YC (entrzID_100281601), WRKY (entrzID_100279570, entrzID_100281558 and entrzID_100285217), were down-regulated by both stresses (Supplementary Tables S5,S7–S9). It suggested these TFs shared downstream pathway of signal transduction and pathway regulation. And the bHLH family considered to be play central roles in responsive to low temperature and high salt and drought, iron and phosphorus deficiency stresses, etc.^51^. NF-YC, NAC and WRKY families are also involved in abiotic stress tolerance in plant, for examples, the overexpression of an *Amaranthus hypochondriacus* NF-YC gene enhances water stress resistance in *Arabidopsis*^47^, the NAC family TFs (such as OsNAP, OsNAC52) confer abiotic stress response through the ABA pathway in rice^52^, WRKY30 had a positive regulatory effect on the growth of grape under salinity stress^53^. Here, NF-YC, NAC and WRKY families were regulated by cold and drought stresses (Supplementary Tables S5,S7–S9), of which entrzID_100273749 (c47289_g2) encoding NAC, entrzID_100285217 (c38115_g1) encoding WRKY, were up-regulated in *Zea mays* ssp. *mexicana* L. variety “8493”under cold stress^44^, suggesting the NAC (entrzID_100273749), WRKY (entrzID_100285217) were important cold responsive genes for maize. However, entrzID_103641134 (Zm00001d024268) encoding NAC, up-regulated in the tolerant maize line YE8112 under drought stress^34^, was down-regulated under cold stress in this study. Additionally, some TFs might play important role in the distinct physiological mechanisms of Zhongdi175 in response to different stresses during seed germination, see Supplementary Table S3. Some TF members have been reported in other plants in response to stress in growth and development. To ARF gene families, Narise et al. (2010) found that IAA14 and ARF7/19 mediated auxin signaling plays an important role in *Arabidopsis* low phosphate adaptation^54^, and Zwiewka et al. (2019) showed that *Arabidopsis* root adaptation to H_2_O_2_-induced oxidative stress is closely related to ARF-GEF BEN1- and cytoskeleton-mediated PIN2 trafficking^55^.

The bZIP and GRAS families are another group of TFs involved in plant tolerance to abiotic stresses, in particular, to cold and drought stresses^12^. And the transcription factor OsABF1 (bZIP), as a binging factor of ABA response element, can enhance abiotic stress signal transduction in rice^56^. SlGRAS40 enhances tomato tolerance to abiotic stress by regulating auxin and gibberellin signal transduction^57^. These results indicated that TFS plays an important role in improving the ability of maize and other crops to resist abiotic stress during growth and development.

### DEGs involved in hormone metabolism and signal transduction

Plant hormones are important growth regulators that regulate plant growth, development, nutrient allocation and source-sink conversion to adapt to stress environment. To date, many studies have shown that different plant hormones, such as ABA, GAs, IAA, CKS, JA, BRs and ETH, control many physiological functions and biochemical processes in plants, including seed germination^18,30,58^.

ABA is the key phytohormone of plants, which regulates gene expression, protein synthesis, signal transduction, and ion transport in response to abiotic stresses such as drought and photoinhibition^59^. Hyperosmotic stress caused by drought or salt stress leads to the accumulation of ABA, and then quickly triggers the downstream response of plants^18,22^. As an example, de Zelicourt et al. (2016) found that cold stress could induce the biosynthesis of ABA, and exogenous ABA can improve the cold tolerance of plants to a certain extent. And previous studies have shown that the bZIP transcription factor OsABF1 is involved in ABA signaling transduction in rice under abiotic stress^56^, NAC family TFs (such as OsNAP, OsNAC54) mediates rice response to abiotic stress through ABA pathway^60^. In the current study, XLOC_046985, entrzID_100501444 (up-regulated under cold stress), entrzID_100501454 (up-regulated under drought stress) and entrzID_732819 (up-regulated under cold and drought stresses) encoding NCED, was identified during Zhongdi175 seed germination (Fig. 6), which is consistent with the results of NCED1 (c63716_g5, up-regulated by drought) reported previously in *Zea mays* ssp. *mexicana* L.^44^ and the results of GRMZM2G407181 (entrzID_100501444) and GRMZM2G110192 (entrzID_100501454) encoding NCED up-regulated under drought stress reported by Kakumanu et al. (2012), suggesting conserved regulation of this drought induced response and ABA-dependent pathways play a role in maize response to drought. Therefore, bZIP/NAC-ABA pathways play critical roles in the response to cold or/and drought stresses during seed germination of Zhongdi175. Our results further indicated that ABA biosynthesis played an important role in plant response to environmental stresses.

Gibberellins (GAs) play an important role in regulating plant growth and development, including seed germination and stem elongation^61^. GAs are also involved in plant tolerance to abiotic stresses, e.g. the effect of exogenous gibberellin (GA4 + 7) on soybean under flooding stress, and found that GAs could improve plant stress resistance during short-term flooding^62^. And GRAS is involved in plant tolerance to abiotic stress and affects auxin and gibberellin signal transduction^57^. Here, two gibberellin 2-beta-dioxygenase genes (entrzID_100273040 and entrzID_100280480), one ent-kaurene oxidase gene (entrzID_103638615), and two gibberellin 20-oxidase genes (entrzID_100283148 and entrzID_100284800) were regulated by cold or/and drought (Fig. 6), which may play a role in the catabolic pathway of gibberellin by 2β-hydroxylation^63^. And a previous study found that, gene expression of GA 2-beta-dioxygenase (c57017_g1, c33506_g1, and c57017_g2), precursor of GAs receptor GID1L2 (c47232_g1) and GA 20-oxidase (c63822_g4, c49254_g1, and c56760_g2) were up-regulated under cold stress^44^, of which entrzID_100284800 (c63822_g4) encoding gibberellin 20-oxidase gene was up-regulated by drought stress in this study. Therefore, GRAS-GA/auxin pathways may play a key role in the respond of Zhongdi175 seed germination to cold or/and drought stresses.

Auxin is another phytohormone, which is the early detection of plant hormones, widely involved in plant growth and development, including plant responses to abiotic stress^64^. Here, five auxin-responsive Aux/IAA genes (entrzID_100193444, entrzID_100274580, entrzID_100283579, entrzID_100285630, and entrzID_100286028) and five auxin-related genes, indole-3-acetaldehyde oxidase (entrzID_542228 and entrzID_542229), 3-hydroxyindolin-2-one monooxygenase (entrzID_100382554), indole-2-monooxygenase (entrzID_100192631), indole-3-glycerol phosphate synthase (entrzID_100286258) and indole-3-glycerol phosphate lyase (entrzID_542117) which may play an important role in stress resistance during Zhongdi175 seed germination. The genes of entrzID_542117, entrzID_100192631 and entrzID_100382554 were up-regulated under cold and drought stresses; the expression of entrzID_100274580 was down-regulated under cold stress; the expression of entrzID_100193444 and entrzID_100285630 was down-regulated under cold and drought stresses; the expression of entrzID_100286028, entrzID_100283579, entrzID_542228, and entrzID_542229 was up-regulated under cold stress; entrzID_100286258 was up-regulated under drought stress (Fig. 6 and Supplementary Tables S7–S9). Auxin/indole-3-acetic acid proteins are widely involved in plant growth and development through auxin signaling pathway^65^. The stress pathway interacts with the auxin gene regulatory network through the transcription of the Aux/IAA genes, which acts as the hub of integrating genetic and environmental information to achieve plant stress resistance^66^. ARF family is a well-known family in the auxin signal transduction pathway, which regulates the transcription of auxin-induced genes. Recent evidence suggests that the ARF family also functions in plant responses to abiotic stresses^67–69^. Thus, the ARF-Aux/IAA pathway may play an important role in response to cold and/or drought stresses in Zhongdi175 seed germination. In grasses, indoles are converted to indolin-2-ones by the P450 enzyme BX2^67^. In this study, genes encoding *cis*-zeatin-O-glucosyltransferase (entrzID_541881 and entrzID_103636476) and cytochrome P450 (entrzID_103630138, entrzID_103635519, entrzID_103640646, and entrzID_103647886) were identified, which were involved in the tolerance of Zhongdi175 to cold and/or drought stresses (Fig. 6 and Supplementary Tables S7–S9). Kudo et al. identified three putative *cis*-zeatin-O-glucosyltransferases in rice, and proposed that *cis*-zeatin activity had a physiological impact on the growth and development of rice. And Magwanga et al. found that knockdown of cytochrome P450 genes Gh_A13G2057 and Gh_D07G1197 might enhance drought and salt stress tolerance in *Gossypium hirsutum*, which is consistent with our results.

### DEGs involved in flavonoid metabolism pathways

Flavonoids are widely distributed in plants and have secondary metabolites with different metabolic functions. The flavonoids-mediated gene transcription changes in rice involve genes regulating cell wall remodeling, redox homeostasis, auxin signaling, and oxidative stress response and so on^70^. Recent studies have shown that flavonoids accumulate at high levels in polar plants to improve the ability of plants to adapt to cold or drought stress^71^. For example, the concentration of flavonoid compounds in *Achillea pachycephala Rech*.f increased with the increase of drought stress duration, and under a certain degree of drought stress can obtain a high level of flavonoids^72^.

The biosynthetic pathways of flavonoids main including 4-coumaroyl CoA ligase (4CL), anthocyanidin synthase (ANS), cinnamate 4-hydroxylase (C4H), chalcone synthase (CHS), chalcone isomerase (CHI), chalcone reductase (CHR), dihydroflavonol-4-reductase (DFR), flavonol synthase (FLS), flavanone 3-hydroxylase (F3H), flavonoid 3',5'-hydroxylase (F3′5'H), isoflavone synthase (IFS), isoflavone 3'-hydroxylase (IF3H), leucoanthocyanidin dioxygenase (LDOX), O-methyltransferase (OMT), phenylalanine ammonia lyase (PAL)^73^. In the present study, 12 putative flavonoid metabolism-related genes might be important roles in responding to cold or/and drought stresses were identified (Fig. 7). The genes entrzID_100282642 and entrzID_100274415 encoding CHS, were down-regulated in Zhongdi175 seedlings under cold and drought stresses; entrzID_100273683 was up-regulated under cold stress, encoding caffeoyl-CoA O-methyltransferase (Fig. 7 and Supplementary Tables S7–S9), which are two key enzymes of the flavonoid/isoflavonoid biosynthesis pathway^74,75^. In particular, the expression of CHS gene is induced in plants by stresses such as UV light, bacterial or fungal infection, and the like^74^. Other nine genes (entrzID_100381820, entrzID_542258, entrzID_109943525, entrzID_100284998, entrzID_100276821, entrzID_100127010, entrzID_542712, entrzID_103653707, and entrzID_103639113), encode PAL, C4H, CHI, LDOX, F3H, F3′H, F3′5′H, respectively (Fig. 7), of which entrzID_100381820 (Zm00001d017279) and entrzID_100274415 (GRMZM2G422750) down-regulated by drought in maize were reported^34,35^. Besides, MYB and/or bHLH TFs always involves regulated the phenylpropanoid or flavonoid biosynthetic pathways^76^. Therefore, MYB-flavonoid or MYB-bHLH-flavonoid biosynthetic pathways may also be important for Zhongdi175 in response to cold or/and drought stresses during seed germination. This study will provide better understanding of the molecular regulation mechanism of flavonoid metabolism in maize seed germination resistance to cold and drought stresses.

### DEGs involved in sucrose metabolism and cell growth promotion

Genes encoding enzymes related to sucrose metabolism, such as galactinol synthase, sucrose synthase, trehalose phosphate phosphatase and trehalose phosphate synthase, were induced under abiotic stresses^18,22^. For example, galactinol synthase genes (*AtGolS1*, *2* and *3*), *AtGolS1* and *2* were induced by drought and high-salinity stresses, while *AtGolS3* was induced by cold stress in *Arabidopsis*^36^; sucrose synthase genes (GRMZM2G089713 and GRMZM2G318780) were up-regulated in ovary of maize inbred line B73 under drought stress^35^; overexpression of trehalose-6-phosphate phosphatase gene *OsTPP1* improved salt and cold tolerance of rice^77^; overexpression of trehalose-6-phosphate synthase gene *OsTPS1* enhanced the tolerance of rice seedlings to abiotic stresses^78^.

Genes encoding fructokinase (entrzID_542108) and hexokinase (entrzID_100170246), which play a role in sugar signaling^79^ and enzymatic function, were regulated under cold and drought stresses in Zhongdi175 seedlings. These genes were also induced by drought stress in maize inbred line B73 ovary^35^. The results show sucrose metabolism is vital for the adaptation of Zhongdi175 to abiotic stresses during seed germination.

Cell-wall metabolism related genes were in response to cold and drought stresses in Zhongdi175 seedlings. For example, beta-expansin 7 (entrzID_542649, Zm00001d029906) was induced in sensitive drought and tolerant drought maize^34^. It is well known that β-expansin is a key regulator of cell wall modification during tissue elongation^80,81^. Overexpression of wheat beta-expansin gene *TaEXPB23* enhances tobacco root growth and water stress tolerance^82^. The cellulose in the primary cell wall, which is laid down during plant growth, determines the shape of the plant^83,84^. Chen et al. (2005) found that disruption of the cellulose synthase gene *AtCesA8/IRX1* was found to enhance the tolerance of *Arabidopsis* to drought and osmotic stresses. In additon, the expression of entrzID_100192868 was up-regulated under cold stress in this study (Supplementary Tables S7–S9), which was consistent with that the expression of entrzID_100192868 was up-regulated in drought-sensitive and drought-tolerant maize^34^. Therefore, sucrose metabolism could help plants to maintain normal growth under stress conditions, and cell wall remodeling can help the cells conserve water, thus contributing to better adaptation to abiotic stresses during maize seed germination.

### Common and unique molecular mechanisms of response to different abiotic stresses in maize

Plants often simultaneously adapt to different adverse environmental conditions and evolve common mechanisms to respond to various abiotic stresses^85^. In our dataset, many up-regulated DEGs were found in both stress samples (cold and drought), however, the functions of most of the down-regulated genes remain to be explored (Supplementary Tables S7–S9). Here, we screen and obtained some important DEGs related to transcriptome factors (Table 2), hormone metabolism and signal transduction, flavonoid metabolism, sucrose metabolism and cell growth promotion and so on. This study laid an important foundation for us to better understand the mechanism of maize seed germination in response to cold and drought stresses. In agricultural production, many kinds of abiotic stresses occur simultaneously^85^. At present, there are some report on that transcriptome of maize plants in response to abiotic stresses. Transcriptome comparative analysis based on different maize materials, different growth stages, different tissues and different adverse environments may play an important role in clarifying some common molecular mechanisms of plant abiotic stress response. For example, Li et al. analyzed the transcriptome of B73 seedling leaves under abiotic stresses, we compared the relevant results of this study with them, and found that GO:0016491 and GO:0005840 were also enrich in B73 leaves under multiple stress conditions (Fig. 4b) and entrzlD_100272864, entrzlD_100217270 and entrzlD_100281366 were also commonly enriched in B73 leaves under abiotic stresses, which involved in abscisic acid, ethylene and jasmonic acid biosynthesis and signal transduction pathways, respectively (Fig. 6). Relevant work needs to be further carried out in the later stage.

## Conclusions

In this study, RNA-Seq was used to detect the whole transcriptional changes of maize seedlings under abiotic stresses during seed germination. Totally, 12,299 DEGs were obtained, with 9605 and 7837 DEGs responding to cold and drought stresses, respectively. Among them, 5143 DEGs were regulated by both stresses, indicating that there were many common and unique molecular mechanisms in the resistance of Zhongdi175 seed germination to different abiotic stresses. Genes related to TFs, hormone metabolism and signaling, flavonoid metabolism, sucrose metabolism and cell growth promotion were found to involve in the resistance to cold and/or drought stresses in Zhongdi175 seedlings during seed germination. Importantly, a total of 2248 transcription factor (TF) genes from 56 TF families were identified; the identified DEGs mainly enriched in ribosome, phenylpropanoid biosynthesis and secondary metabolites biosynthesis pathways under and/or drought stresses. Furthermore, some important genes and pathways have been found, such as sucrose metabolism and cell growth promotion genes, MYB-flavonoid or MYB-bHLH-flavonoid biosynthetic pathways, ARF-Aux/IAA, bZIP/NAC-ABA and GRAS-GA/auxin pathways, etc. This research expanded the understanding of the molecular mechanism of high vigour maize seed germination (seedling establishment) stress resistance, and provided the basis for the selection of major candidate genes and molecular markers of maize stress resistance and the breeding of high vigour maize cultivars.

## Supplementary Information

Supplementary Figure 1.

Supplementary Tables.

Supplementary Legends.
