# Supplementary figures and images for: Transcriptomic profiling of the high-vigour maize (Zea mays L.) hybrid variety response to cold and drought stresses during seed germination
Source: Sci Rep. 2021 Sep 29;11:19345. doi: 10.1038/s41598-021-98907-8 (PMC8481303; doi:10.1038/s41598-021-98907-8)

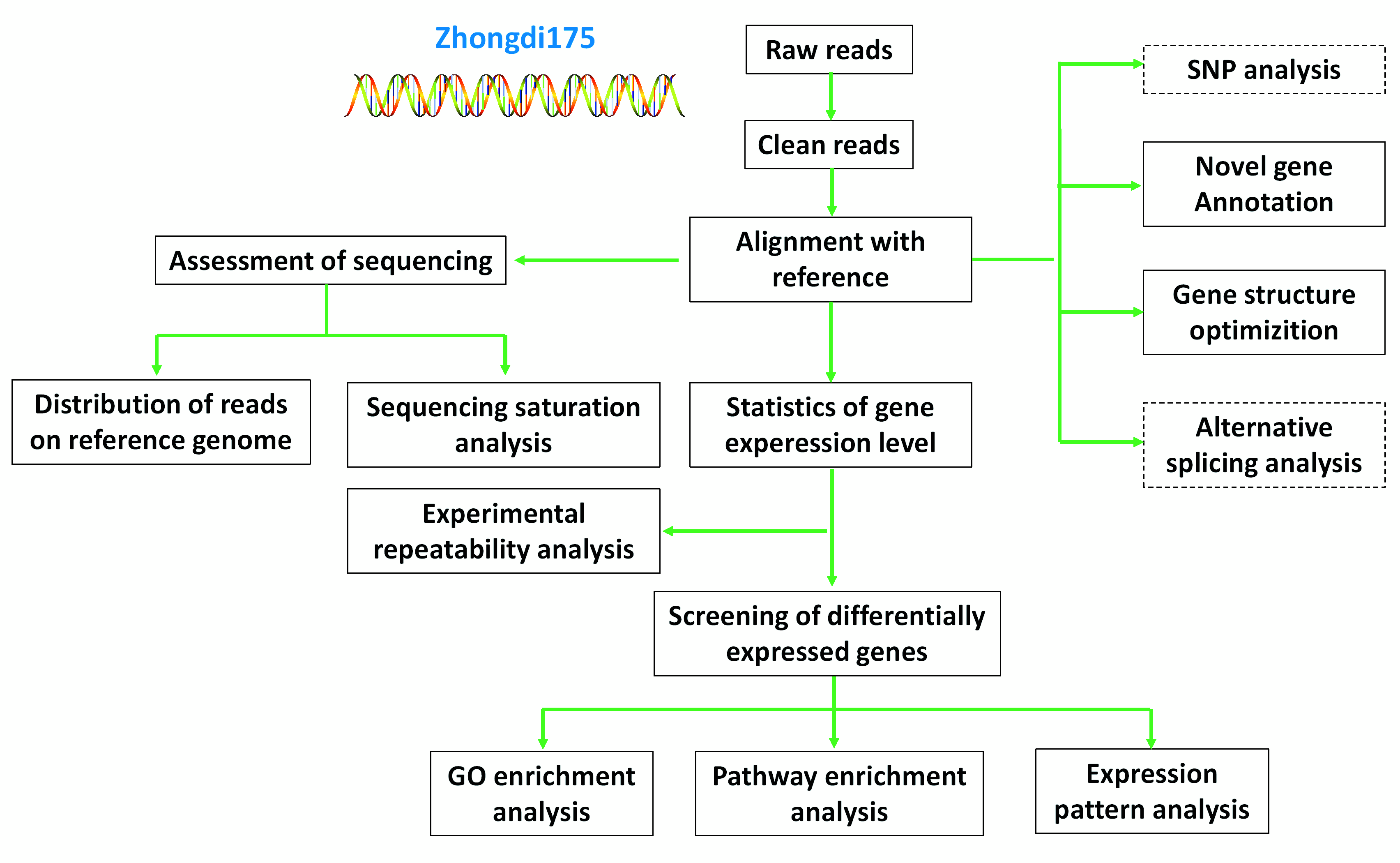

Supplement: Supplementary file 1 — Supplementary Figure 1. [file 41598_2021_98907_MOESM1_ESM.tif]
