# Supplementary material for: Transcriptomic profiling of the high-vigour maize (Zea mays L.) hybrid variety response to cold and drought stresses during seed germination
Source: Sci Rep. 2021 Sep 29;11:19345. doi: 10.1038/s41598-021-98907-8 (PMC8481303; doi:10.1038/s41598-021-98907-8)
Supplement: Supplementary file 3 — Supplementary Legends. [file 41598_2021_98907_MOESM3_ESM.docx]

**Additional Information**

Supplementary Table S1. Primers used in the qRT-PCR assay.

Supplementary Table S2. GO items related to cold and drought resistance of Zhongdi175 seed germination.

Supplementary Table S3. Important transcription factors involved in Zhongdi175 seedlings response to abiotic stresses during seed germination.

Supplementary Table S4. Expression quantification of all genes (FPKM > 0 from at least one treatment) of Zhongdi175 after screening.

Supplementary Table S5. Transcription factor (TF) genes identified in the Zhongdi175 transcriptome.

Supplementary Table S6. Expression quantification of all genes (FPKM ≥ 1 from at least one treatment) of Zhongdi175 after screening.

Supplementary Table S7. Significantly DEGs by direct comparing cold stress with control.

Supplementary Table S8. Significantly DEGs by direct comparing drought stress with control.

Supplementary Table S9. Significantly DEGs by direct comparing drought with cold stresses.

Supplementary Table S10. GO enrichment analysis of DEGs by direct comparing cold stress with control.

Supplementary Table S11. GO annotation of DEGs by direct comparing cold stress with control.

Supplementary Table S12. GO enrichment analysis of DEGs by direct comparing drought stress with control.

Supplementary Table S13. GO annotation of DEGs by direct comparing drought stress with control.

Supplementary Table S14. GO enrichment analysis of DEGs by direct comparing drought with cold stresses.

Supplementary Table S15. GO annotation of DEGs by direct comparing cold stress with drought stress.

Supplementary Table S16. KEGG enrichment analysis of DEGs by direct comparing cold stress with control.

Supplementary Table S17. KEGG enrichment analysis of DEGs by direct comparing drought stress with control.

Supplementary Table S18. KEGG enrichment analysis of DEGs by direct comparing cold stress with drought stress.

Supplementary Figure S1. Flow chart of RNA-seq data analysis.
